# Genetic Characteristics and Pathogenicity of a Novel Porcine Deltacoronavirus Southeast Asia-Like Strain Found in China

**DOI:** 10.3389/fvets.2021.701612

**Published:** 2021-07-16

**Authors:** Hejie Wang, Yibin Qin, Wu Zhao, Tingting Yuan, Chunjie Yang, Xue Mi, Ping Zhao, Ying Lu, Bingxia Lu, Zhongwei Chen, Ying He, Cui Yang, Xianfeng Yi, Zhuyue Wu, Ying Chen, Zuzhang Wei, Weijian Huang, Kang Ouyang

**Affiliations:** ^1^College of Animal Science and Technology, Guangxi University, Nanning, China; ^2^Department of Virology, Guangxi Veterinary Research Institute, Nanning, China; ^3^Guangxi Key Laboratory of Livestock Genetic Improvement, Guangxi Institute of Animal Science, Nanning, China

**Keywords:** porcine deltacoronavirus, virus isolation, diarrhea, genomic characteristics, pathogenicity, piglets

## Abstract

Farmers involved in the lucrative pork trading business between China and Southeast Asian countries should be aware of a recently discovered novel porcine deltacoronavirus (PDCoV) in Guangxi province, China. A PDCoV strain, CHN/GX/1468B/2017, was isolated from the small intestinal contents of piglets with diarrhea from this region, with a titer of 1 × 10^8.0^ TCID_50_/mL on LLC-PK cells. The full-length genome sequence consists of 25,399 nt as determined by next-generation sequencing and this was deposited in the GenBank (accession number MN025260.1). Genomic analysis showed that CHN/GX/1468B/2017 strain had 96.9~99.4% nucleotide homology with other 87 referenced PDCoV strains from different areas, and contained 6 and 9-nt deletions at positions 1,733~1,738 and 2,804~2,812, respectively, in the ORF1a gene. Phylogenetic analyses based on the whole gene sequence as well as S protein and ORF1a/1b protein sequences all showed that this strain was closely related to the Southeast Asia strain. When 7-day-old piglets were inoculated orally with the CHN/GX/1468B/2017 strain, they developed severe diarrhea, with a peak of fecal viral shedding at 4 days post-infection. Although no death or fever were observed, the CHN/GX/1468B/2017 strain produced a wide range of tissue tropism, with the main target being the intestine. Importantly, the VH:CD ratios of the jejunum and ileum in infected piglets were significantly lower than controls. These results indicate that CHN/GX/1468B/2017, isolated in China, is a novel PDCoV Southeast Asia-like strain with distinct genetic characteristics and pathogenicity. This finding enriches the international information on the genetic diversity of PDCoV.

## Introduction

In recent years, enteric coronaviruses have been deemed to be one of the most serious pathogens that can endanger the healthy development of the farmed pig. Examples include PEDV (1), TGEV (2), swine acute diarrhea syndrome coronavirus (SADS-CoV) also called swine enteric alphacoronavirus (SeACoV) and porcine deltacoronavirus (PDCoV) (3). PDCoV is an enveloped, single-stranded positive-sense RNA virus (4), which belongs to the genus *Deltacoronavirus* of the *Coronaviridae* family. PDCoV causes acute diarrhea, vomiting, dehydration and even death in suckling piglets (5, 6). Clinical reports indicated that the pathological lesions in the jejunum and ileum from PDCoV infected piglets were characterized by severe atrophy and inactivation of villi as well as swelling of villous cells (5, 7, 8).

The full-length genome of PDCoV is ~25.4 kb. PDCoV has seven main open reading frames (ORFs). ORF1a/1b encode polyproteins pp1a and pp1ab and subsequently auto-proteolyzed into 15 non-structural proteins (Nsp2 to Nsp16). The remaining ORFs encode at least four structural proteins: spike protein (S), envelope protein (E), membrane protein (M), nucleocapsid protein (N) (9). Among these, the S protein interacts with the host cell receptor to mediate fusion of the virus envelope with the host cell membrane, which is a crucial step for virus entry into cells (10). E and M are transmembrane proteins which are important for virion assembly and budding (11, 12). The N protein is highly conserved and plays a key role in the encapsulation of viral RNA (13, 14).

PDCoV has been shown to have broad cellular tropism. It can replicate in multiple host cells, including porcine, humans and chicken cells (15), but LLC-PK may be the ideal cell line for PDCoV infection (16). To date, PDCoV has been reported in the United States (17), Canada (18), Mexico (19), Thailand (20), Laos (21), South Korea, and China, and exhibits a global distribution trend with accompanying economic losses to the swine industry. From 2012 to 2018, the positive rate of PDCoV in the diarrhea samples collected from pigs in different parts of China was between 19.4 and 36.18% (22–25). So far, PDCoV is still one of the major viruses that endanger the swine industry in China.

High virus genetic diversity and frequent recombination have been identified in PDCoV (26). Previous studies have shown that the genetic diversity of PDCoV strains from Vietnam was influenced by introduction of strains from neighboring countries, and the viral PDCoV strains from Thailand, Laos, and Vietnam appeared to cluster into a new group, subsequently known as the Southeast Asia (SEA) strain (27, 28). The southwestern province of Guangxi is one of major pig-breeding regions of China and it is adjacent to the countries of Southeast Asia. In recent years, there has been increasing trade between China and these countries. However, little is known regarding the information of PDCoV found in the Guangxi region. The objective of this study was to investigate the genetic characteristics and pathogenicity of a novel PDCoV Southeast Asia-like strain found in Guangxi, China.

## Materials and Methods

### Collection and Treatment of Clinical Samples

Small intestinal contents and feces were collected from suckling piglets with diarrhea in Guangxi, China. Clinical samples were homogenized with phosphate-buffered saline (PBS) at a ratio of 1:10. Supernatants were collected by centrifugation at 12,000 rpm/min for 30 min at 4°C, and these were filtered through 0.22 μm filters (Millipore, Germany) and stored at −80°C. The filtered supernatants were used as the inoculum for virus isolation.

### RT-PCR

Viral RNA was extracted from sample suspensions using the Axy Prep^TM^ Viral DNA/RNA Miniprep Kit (OMEGA, USA) according to the manufacturer's instructions. Complementary DNA (cDNA) was synthesized using oligo-dT in a 25 μL reaction mixture containing 5 μL of 5 × Reversa Transcriptase M-MLV Buffer, 2 μL of dNTP mixture, 1 μL of Oligo dT, 0.5 μL of M-MLV reverse transcriptase, 0.5 μL of ribonuclease inhibitor, 16 μL of RNA template and were incubated at 42°C for 60 min. The PCR reaction was carried out in a total volume of 25 μL containing 12.5 μL of 2× Taq PCR Master Mix, 0.5 μL of each primer, 3 μL of cDNA template and 8.5 μL of nuclease-free H_2_O. Thermal cycles consisting initial denaturation at 95°C for 3 min, followed by 35 cycles at 94°C for 40 s, 56°C or 51°C for 40 s, and 72°C for 60 s and then final extension at 72°C for 10 min. Primers used for PCR of PDCoV, PEDV, TGEV, and porcine rotavirus (PoRV) are listed in Supplementary Table 1.

### Virus Isolation

LLC porcine kidney cells (LLC-PK) were purchased from the American Type Culture Collection (ATCC, No. CL-101) and cultured in high glucose Dulbecco's Modification of Eagle's Medium (DMEM, Biological Industries, USA) containing 10% fetal bovine serum (FBS, Biological Industries, USA) and 1% antibiotic-antimycotic (Coring, USA). The maintenance medium used for PDCoV propagation was DMEM supplemented with 5 μg/mL trypsin (Gibco, USA).

To isolate PDCoV, 100 μL of the filtered supernatants from PDCoV-positive samples and 400 μL of maintenance medium were inoculated into a 6-well-cell culture plate with containing LLC-PK cells monolayers grown to 80~90% confluence. After absorption for 1.5 h in an incubator filled with 5% CO_2_ at 37°C, the cells were washed three times with PBS. Two milliliters of maintenance medium were added to the cells, which were cultured until the cells showed cytopathic effects (CPEs). Then the cell plate was frozen and thawed twice at −80°C. The supernatants were collected and stored at −80°C. The collected cultures from each generation of cells were subjected to RT-PCR and indirect immunofluorescence assay (IFA) identification. The isolated PDCoV strain was designated as CHN/GX/1468B/2017, and the titers for each batch (TCID_50_) were determined.

### Indirect Immunofluorescence Assay

LLC-PK cell monolayers in 6-well cell plates were incubated with the PDCoV CHN/GX/1468B/2017 strain at a multiplicity of infection (MOI) of 0.01 for 24 h. The cells were fixed with 4% paraformaldehyde for 30 min and treated with 0.2% Triton X-100 (Sigma, USA) for 20 min at room temperature. After being washed three times with PBS, the cells were blocked with 5% skimmed milk powder for 1 h at room temperature. Subsequently, cells were incubated with mouse-derived PDCoV-N protein-specific polyclonal antibody (1:200) for 1 h at 37°C. Then the cells were washed with PBS three times followed by incubation with FITC-labeled goat anti-mouse IgG (KPL, USA) (1:1,000) for 1 h at 37°C. Finally, images were captured using an inverted fluorescence microscope (Nikon, Tokyo, Japan).

### Genetic Evolution and Mutation Analysis

The full-length genome sequence of the PDCoV CHN/GX/1468B/2017 strain at passage 4 (10^8.0^TCID_50_/mL) was determined by next-generation sequencing (NGS) technology using an Illumina MiSeq 250PE/300PE platform (GENEWIZ, Inc, USA). Sequences were mapped to the PDCoV prototype strain CHN/HKU15-44/2009 and were assessed using the quality control tool.

Based on comparison of the whole genome sequence of the PDCoV CHN/GX/1468B/2017 strain with the other 87 available strains deposited in the GenBank (Supplementary Table 2), sequence alignment was performed using EditSeq and MegAlign software in DNAStar Lasergene (Version 7). Based on the sequences of the complete genome and the S protein and ORF1a/1b protein sequences, phylogenetic trees were constructed, respectively, by using the neighbor-joining method in MEGA6.0 software with a bootstrap of 1,000 replicates, and these visualized with iTOL (https://itol.embl.de/) (29). Additionally, the possible recombination events of the CHN/GX/1468B/2017 strain were predicted by using Recombination Detection Program 4 (RDP4) and Simplot 3.5.1 software packages (30).

### TaqMan Probe Fluorescence Real-Time Quantitative RT-PCR

RNA extraction was performed as described above and then RNA was used for performing qRT-PCR using specific primers and probes with minor modifications (6). A standard curve was generated by using 10-fold serially diluted samples of positive recombinant plasmids. Briefly, 2 μL RNA was used in a 20 μL PCR reaction system consisting of the Transcript Probe One-step qRT-PCR Super Mix kit (Tiangen, China) by using an Applied Biosystems 7500 Quantstudio 3,815 real-time quantitative PCR instrument (Life Technology, USA) under the following conditions: one cycle at 95°C for 5 min followed by 45 cycles at 94°C for 5 s and 60°C for 30 s. The viral RNA load in each sample was calculated based on Ct-values obtained from the constructed standard curves.

### Pathogenicity of PDCoV CHN/GX/1468B/2017 Strain in Piglets

In order to determine the pathogenicity of the PDCoV CHN/GX/1468B/2017 strain in piglets, challenge experiments were performed. The animal study was reviewed and approved by the Animal Care and Welfare Committee of Guangxi University. Eight seven-day-old healthy piglets were purchased from a Guangxi conventional commercial pig farm and were randomly divided into two groups of four and these were housed in separate animal rooms. Fecal samples from all the piglets were confirmed to be negative for PDCoV, PEDV, TGEV, PoRV, African classical swine fever virus (ASFV), porcine reproductive and respiratory syndrome virus (PRRSV), porcine pseudorabies (PRV), and porcine circovirus type 2 (PCV2) by RT-PCR. All piglets were artificially fed a mixture of lure milk (Shenzhen Premix Inve Nutrition Co., Ltd., China) with warm water, four times a day. After 24 h of acclimation, each piglet in the inoculated group (*n* = 4) was orally inoculated with the CHN/GX/1468B/2017 strain at a titer of 1.0 × 10^8.0^ TCID_50_/mL (2 mL per pig) as previously described (31, 32), while those in negative control group (*n* = 4) were inoculated with maintenance medium (2 mL per pig).

Clinical symptoms including vomiting, diarrhea, drowsiness and loss of appetite were observed and recorded daily in all piglets. The change in body temperature and weight of the piglets were measured once a day. The severity of diarrhea for each piglet was scored using the following criteria: 0 = normal, 1 = fecal deformity, 2 = mild diarrhea, and 3 = watery diarrhea. Rectal, oral and nasal swabs were collected daily from each pig and swabs were immersed into 4 mL PBS immediately after collection.

All piglets in each group were euthanized at 7 days post-inoculation (DPI) as previously described (7, 8). Serum and tissue samples including duodenum, jejunum, ileum, cecum, colon, heart, liver, spleen, lung, kidney, stomach, mesenteric lymph nodes, and tonsils were collected. 0.16 g of various tissue samples were mechanically homogenized in 1.6 mL of PBS and centrifuged at 12,000 rpm/min for 5 min at 4°C and supernatants were aliquoted into 1.5 mL Eppendorf tubes. Due to the high sensitivity and specificity, qRT-PCR assay was used to detect viral RNA as previously described (6) with a few modifications.

### Histology and Immunohistochemistry

During necropsy at 7 DPI, tissue samples including duodenum, jejunum, ileum, cecum, colon, heart, liver, spleen, lung, kidney, stomach, mesenteric lymph nodes and tonsils from piglets in both the CHN/GX/1468B/2017 and negative control groups were collected and fixed in 10% formalin for 24 h at room temperature. Then tissues were dehydrated, embedded in paraffin, sectioned and mounted onto glass slides. After staining with hematoxylin and eosin (H&E), the slides were examined and analyzed with a conventional microscope.

Five intestinal villi from each intestinal section were selected randomly and the ratios of villous height and crypt depth (VH:CD) were measured by using Image-Pro Plus 6.0 (Media Cybemetics, USA). Then tissue sections were deparaffinized, rinsed, and antigen repaired with citric acid antigen repair buffer (PH6.0) (Wuhan Servicebio Technology Co., Ltd., China). Bovine serum albumin was used to block non-specific antigens by incubation at room temperature for 30 min. Then, PDCoV-N protein-specific polyclonal antibody was used as the primary antibody diluted at 1:200 ratio by incubating overnight at 4°C. After washing with PBS, the sections were incubated with horseradish peroxidase-labeled goat anti-mouse IgG secondary antibody (1:200) (Dako, China) for 1 h at room temperature. The samples were finally visualized with a 3,3′-diaminobenzidine (DAB) chromogen kit (Wuhan Servicebio Technology Co., Ltd., China).

### Statistical Analysis

Data were expressed as the means ± standard deviations (SDs) of four piglets. Statistical analysis was performed by a two-tailed *t*-test (GraphPad Prism 5.0 software). Values of *p* < 0.05 and 0.01 were considered statistically significant and extremely significant, respectively (33).

## Results

### Virus Isolation and Identification

One strain of PDCoV, named CHN/GX/1468B/2017, was isolated from LLC-PK cells incubated with a sample of small intestinal content collected from a swine farm in Guangxi, with a titer of 1.0 × 10^8.0^ TCID_50_/mL. Obvious CPEs were observed from fourth passage cells at 24 h post inoculated (HPI). Compared with the mock-inoculated cells, the cells inoculated with PDCoV appeared enlarged, rounded and aggregated and eventually died. The virus isolated from the cells were identified by RT-PCR and IFA. Positive PCR results were obtained for PDCoV but they were negative for PEDV, TGEV, and PRoV. Moreover, PDCoV-specific immunofluorescence was detected (Supplementary Figure 1).

### Genomic Characterization of the PDCoV CHN/GX/1468B/2017 Strain

The sequence splicing results showed that the entire genome length of the PDCoV CHN/GX1468B/2017 strain was 25,399 nt, excluding the poly(A) tail, and the details were deposited in the GenBank under accession number MN025260.1. Like other PDCoV strains, CHN/GX1468B/2017 exhibited a classical porcine coronavirus gene structure: 5'UTR-ORF1a/1b-S-E-M-NS6-N-NS7-NS7a-3′UTR corresponding to nucleotide numbers 538, 18,787, 3,482, 251, 653, 284, 1,028, 602, 301, and 385, respectively.

The complete genome of the CHN/GX/1468B/2017 strain shared 96.9~99.4% nucleotide identities with the other 87 PDCoV strains isolated from different areas, exhibiting the highest nucleotide homology (98.3~99.4%) with strains from Southeast Asia. Sequence comparisons indicated that the nucleotide and amino acid homologies of the S gene in the CHN/GX/1468B/2017 strain and Southeast Asia strains were, respectively 96.7~98.9 and 97.7~99.1%, whereas the identity with regards to the CHN/GX/1468B/2017 and China strains were 95.7~96.9 and 96.0~98.1%, respectively. Sequence analysis based on ORF1a gene at the nucleotide and amino acid levels, showed that the CHN/GX/1468B/2017 strain had 98.8~99.4 and 99.1~99.4% similarity with Southeast Asia strains, and shared 96.8~98.0 and 97.7~98.8% similarity with China strains, respectively (Supplementary Table 3).

**Figure 1:**
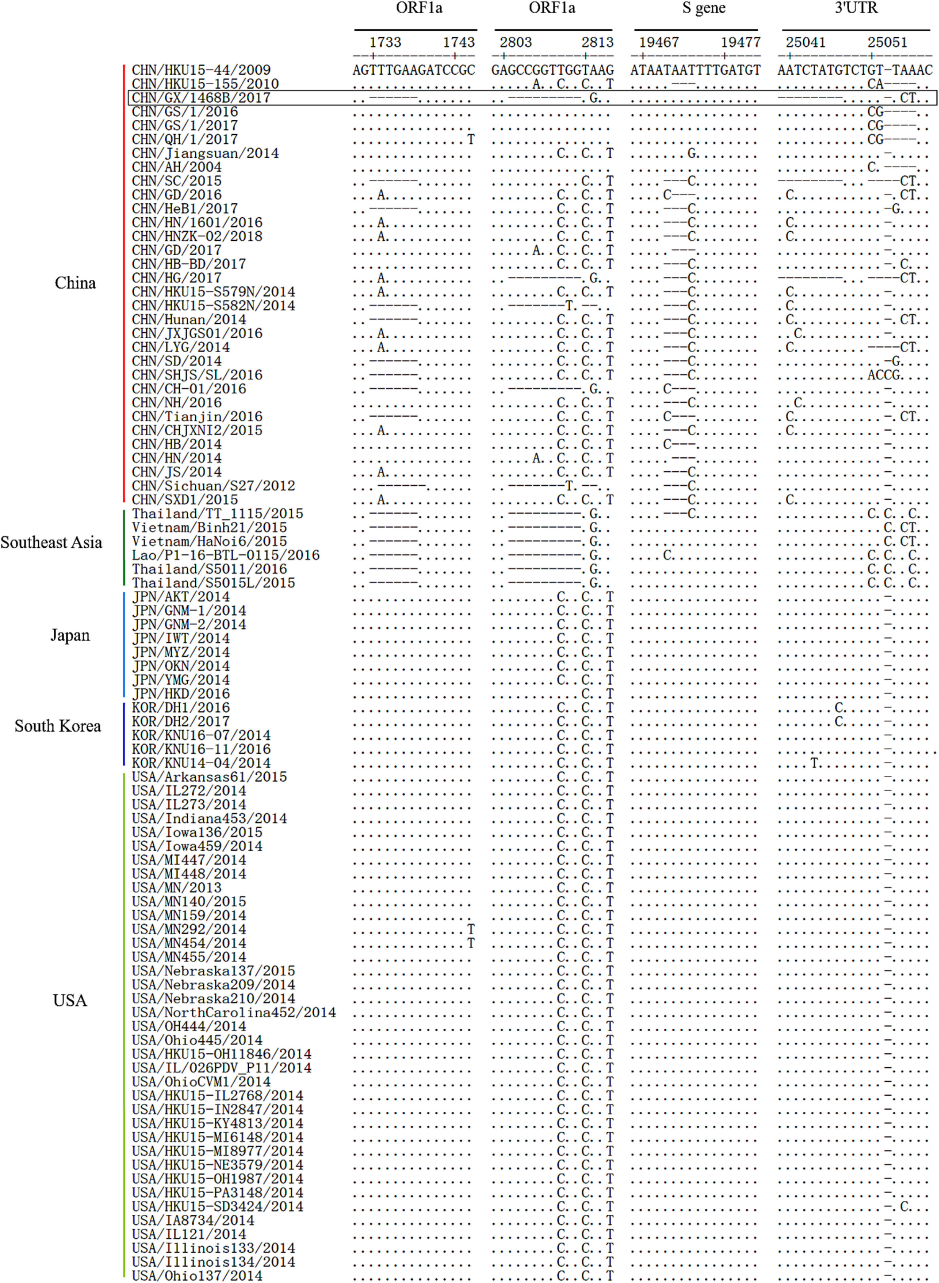
Genome deletions or insertions identified in the complete sequence alignment of PDCoV strains. Sequence alignments of PDCoV strains from different countries by using EditSeq and MegAlign in DNAStar Lasergene Version 7 software packages. The dots (•) and dashes (–), respectively, indicate the exact nucleotide matches or deletions compared to the CHN/HKU15-44/2009 strain, while the boxes indicate the main deletions and insertions in the PDCoV CHN/GX/1468B/2017 strain.

Compared to the PDCoV strains, CHN/HKU15-44/2009 and CHN/HKU15-155/2010 which were originally identified in Hong Kong, the aligned sequence of the CHN/GX/1468B/2017 strain contained 6-nt (TTTGAA) and 9-nt (GCCGGTTGG or GCCAGTCGG) deletions at positions of 1,733~1,738 and 2,804~2,812 in the ORF1a gene, respectively. However, these deletions only existed in all the referenced Southeast Asia PDCoV strains, and did not exist in the Japan, South Korea, and USA strains. There was a 3-nt (TAA) deletion in the S gene of most China strains at position 19,471~19,473, but this was not seen in the CHN/GX/1468B/2017 strain. In addition, the CHN/GX/1468B/2017 strain has a continuous deletion of 8-nt (AATCTATG) at position 25,040~25,047 in the 3′UTR region which was also found in strains CHN/HG/2017 and CHN/SC/2015 from China. This deletion was not found in the other 85 strains of PDCoV (Figure 1). No recombinant event was found in the CHN/GX/1468B/2017 strain when analyzed using the RDP4 and Simplot 3.5.1 software packages.

### Phylogenetic Analysis of the PDCoV CHNN/GX/1468B/2017 Strain

**Figure 2:**
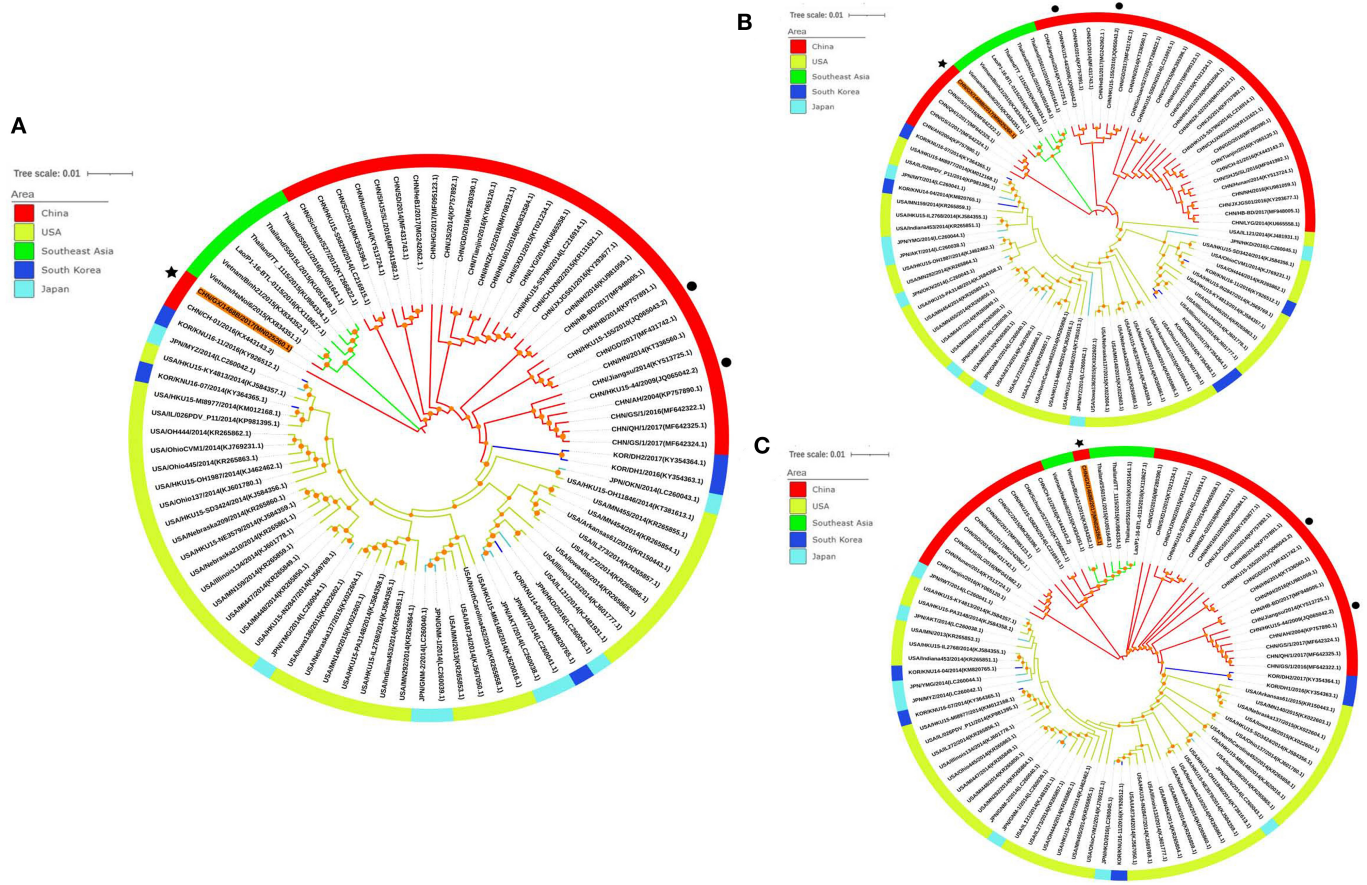
Phylogenetic trees of PDCoV strains based on the sequences of the complete genome **(A)** and the S protein **(B)** and ORF1a/1b proteins **(C)**. Phylogenetic trees were constructed using the neighbor-joining method in MEGA6 software and a bootstrap analysis was performed with 1,000 replicates. The trees were visualized using the online software, iTOL (https://itol.embl.de/), and bootstrap values >50% are shown at the branch points. Each PDCoV strain is indicated in the following format: country origin (CHN, China; JPN, Japan; KOR, South Korea; USA, the United States; Lao; Thailand and Vietnam)/strain name/year of collection (GenBank accession number). The CHN/GX/1468B/2017 strain obtained in this study is indicated with a black asterisk, the CHN/HKU15-44/2009 and CHN/HKU15-155/2010 strains originally identified in Hong Kong are indicated with a black dot. The branches in red, green, yellow, light blue, and dark blue represent the clades from China, Southeast Asia, USA, Japan, and South Korea, respectively.

Phylogenetic analysis trees were constructed based on the whole gene sequence as well as the S protein and ORF1a/1b protein sequences of the PDCoV CHN/GX/1468B/2017 strain and the 87 referenced strains in GenBank, respectively. The phylogenetic analysis of the complete genome showed that the PDCoV CHN/GX/1468B/2017 strain and strains from Southeast Asia were clustered into a subcluster. The genetic similarity of the CHN/GX/1468B/2017 strain was close to the Southeast Asia strains of Vietnam/HaNoi6/2015 and Vietnam/Binh21/2015. Phylogenetic trees based on the amino acid sequences of S protein and ORF1a/1b both showed that the CHN/GX/1468B/2017 strain and strains from Southeast Asia were all clustered into a large clade, and displayed a very close phylogenetic relationship to these two Vietnamese strains (Figure 2).

### Clinical Symptoms of Piglets Challenged With the PDCoV CHN/GX/1468B/2017 Strain

**Figure 3:**
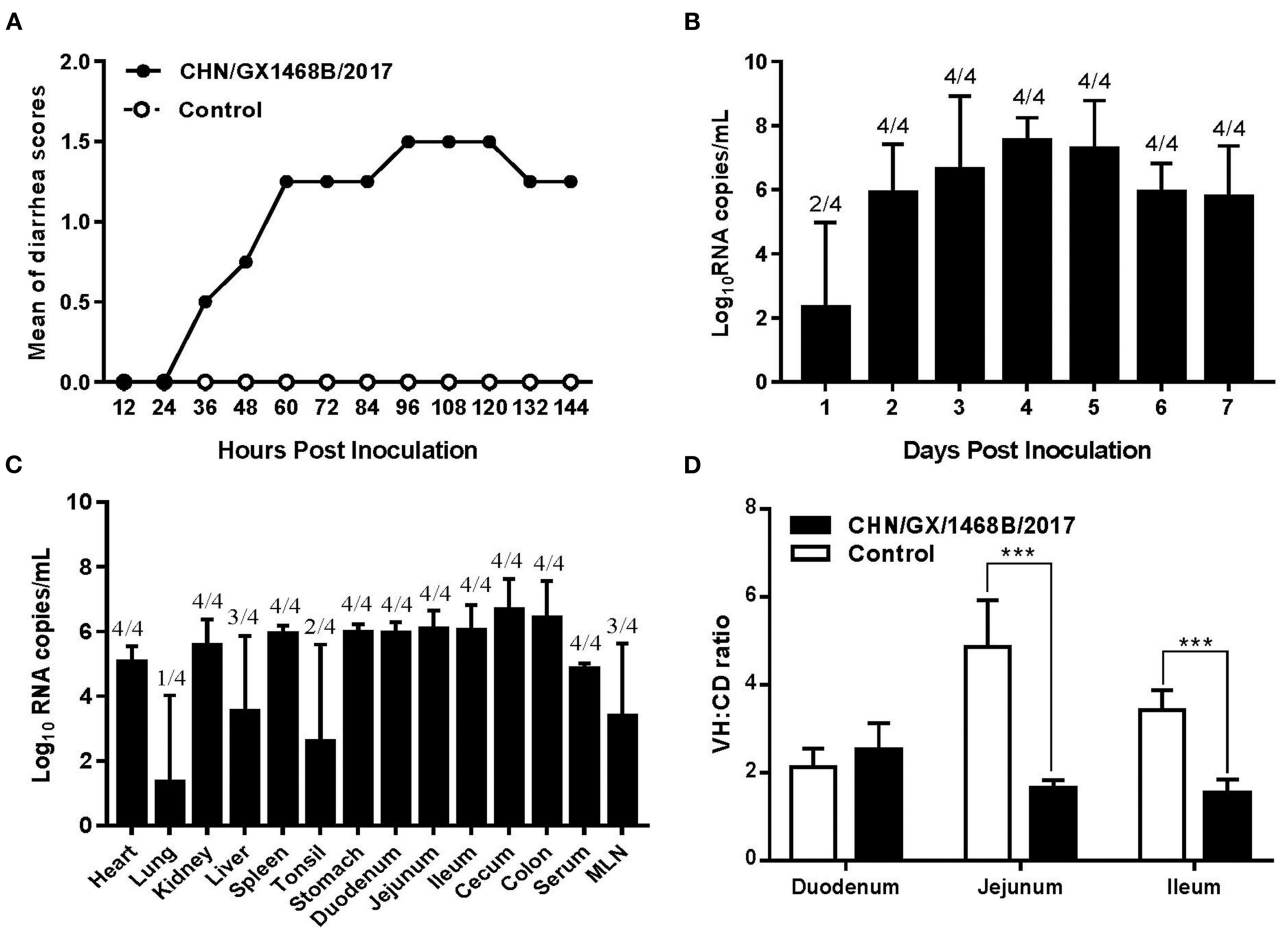
Diarrhea scores, fecal viral shedding, virus distribution and the VH:CD ratios in piglets infected with the PDCoV CHN/GX/1468B/2017 strain. **(A)** Diarrhea scores of 7-day-old piglets challenged with the PDCoV CHN/GX/1468B/2017 strain. Clinical symptoms were monitored daily, and the severity of diarrhea for each piglet was scored using the following criteria: 0 = normal, 1 = fecal deformity, 2 = mild diarrhea, and 3 = watery diarrhea. **(B)** Fecal viral shedding in 7-day-old piglets challenged with the PDCoV CHN/GX/1468B/2017 strain. PDCoV RNA shedding in the feces was quantitated daily from the four challenged piglets. **(C)** Virus distribution in 7-day-old piglets challenged with the PDCoV CHN/GX/1468B/2017 strain. PDCoV RNA loads in tissues collected at 7 DPI were measured by using qRT-PCR. **(D)** The ratio of villous height to crypt depth (VH:CD) in small intestines from piglets inoculated with the PDCoV CHN/GX/1468B/2017 strain. Each point/bar represents the average values of four piglets, the numbers above the bar in graphs **(B,C)** represent the ratio of PDCoV positive piglets to the total in each group, and the asterisk indicates a statistically significant difference between the negative control and CHN/GX/1468B/2017 groups (***p < 0.001).

To evaluate the pathogenicity of the PDCoV CHN/GX/1468B/2017 strain, 7-day-old piglets were orally inoculated with 2 mL of 1.0 × 10^8.0^ TCID_50_/mL virus particles. During the experiment, no drowsiness, fever, loss of appetite, or death was observed in any of the animals. Three of the four infected piglets developed diarrhea and vomiting at 36~144 HPI. The fecal consistency was scored, the average diarrhea score was 0.5~1.25 at 36~84 HPI which reached a peak at 96~120 HPI with a score of 1.5 (Figure 3A). The weight gain ratio of piglets in the challenged group were always lower than that of the negative control group, but not significant (Supplementary Figure 2). No any clinical symptoms were observed in the negative control group of piglets.

### Fecal Shedding and Virus Distribution in PDCoV-Inoculated Piglets

The fecal virus shedding in piglets infected with the PDCoV CHN/GX/1468B/2017 strain was determined by qRT-PCR. In this study, PDCoV RNA (average 10^2.31^ copies/mL) was detected in rectal swab samples from 2 piglets at 1 DPI. Subsequently, all fecal samples were PDCoV positive until the end of the experiment. PDCoV viral RNA peaked on 4 DPI with an average of 10^7.52^ copies/mL (Figure 3B). In addition, the highest amount of viral RNA copies were 10^6.65^ copies/mL and 10^5.50^ copies/mL in nasal swabs at 4 DPI and oral swabs at 5 DPI, respectively.

On autopsy at seven DPI, the virus distribution in various tissues was detected (Figure 3C). Among them, viral RNA could be detected in 1/4 lungs (10^5.57^ copies/mL), 2/4 tonsils (average 10^2.59^ copies/mL), 3/4 livers (average 10^3.52^ copies/mL), and 3/4 mesenteric lymph nodes (average 10^3.38^ copies/mL) from piglets infected with the CHN/GX/1468B/2017 strain. Moreover, viral RNA could also be detected in the hearts, spleens, kidneys, duodenum, jejunum, ileum, cecum, colon, and serum from all four infected piglets, with the average values of 10^4.85^~10^6.67^ copies/mL. In addition, the average viral RNA copies in cecum and colon was as high as 10^6.67^ copies/mL and 10^6.42^ copies/mL, respectively. No PDCoV RNA was detected in any swabs and tissues from piglets in the negative control group.

### Gross Pathology, Histopathology, and Immunohistochemistry in Piglets Infected With the PDCoV CHN/GX/1468B/2017 Strain

**Figure 4:**
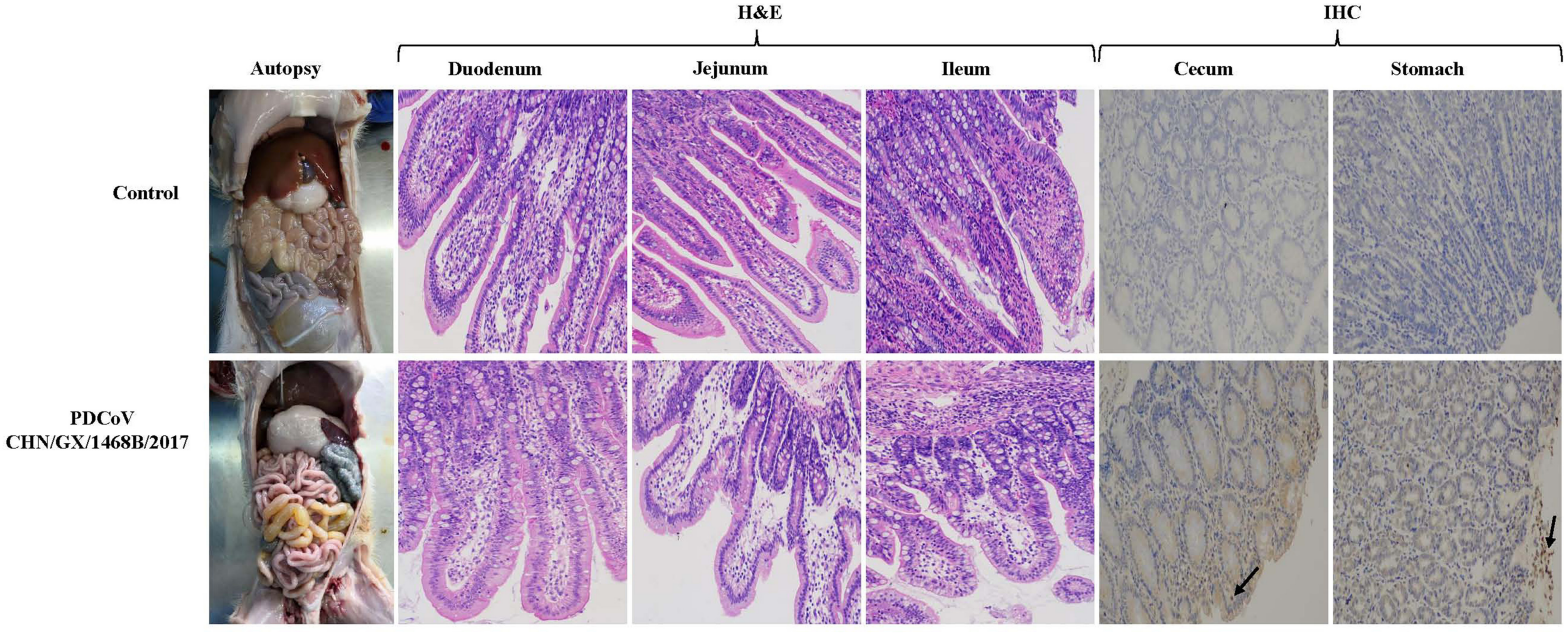
Gross pathological, histopathological and immunohistochemical staining in piglets infected with the PDCoV CHN/GX/1468B/2017 strain. Typical macroscopic intestinal lesions were observed in piglets infected with the PDCoV CHN/GX/1468B/2017 strain by necropsy examination. The small intestine was expanded, filled with yellow water-like liquid and the intestinal wall became thin and transparent. H&E staining showed severe intestinal damage in infected piglets, especially in the small intestine. Obvious visible lesions were observed in the jejunum and ileum, and the villi from these regions were severely atrophied and blunted. In addition, the superficial villi on the epithelial cells were swollen and vacuolated. The duodenal cells were swollen and vacuolated. PDCoV antigen was detected by IHC using PDCoV-N protein specific polyclonal antibody and HRP-labeled goat anti-mouse IgG antibody. PDCoV antigen signals with brown coloring were detected in the cecum and stomach of piglets infected with the CHN/GX/1468B/2017 strain (indicated with black arrows). No antigen was detected in control piglets. (200× magnification).

Macroscopic examination of all piglets infected with the PDCoV CHN/GX/1468B/2017 strain exhibited certain specific symptoms. Their small intestines became distended, thin and transparent and were filled with a large amount of yellow watery content (Figure 4). Obvious histologic lesions were observed in the duodenum, jejunum and ileum of infected piglets. The villi of the small intestine were severely atrophied and blunted. In addition, the superficial villi on the epithelial cells were swollen and vacuolated. Importantly, The VH:CD ratios of the jejunum (1.63 ± 0.17) and ileum (1.53 ± 0.27) from piglets inoculated with the CHN/GX/1468B/2017 strain were both very significantly lower than negative controls (jejunum: 4.85 ± 0.96; ileum: 3.42 ± 0.41; *p* < 0.001), respectively (Figure 3D).

The expression of PDCoV antigen in tissues of piglets challenged with the CHN/GX/1468B/2017 strain was observed by immunohistochemistry. PDCoV antigens could be detected in the cecum and stomach, but the signals were not strong (Figure 4), while no visible PDCoV antigen was detected in any tissues of the negative control piglet group.

## Discussion

As an important determinant in the biological characteristics of PDCoV is that the S gene is not only closely related to gene mutation and virulence of the virus, but also closely relevant to the viral tissue tropism it causes (34). In this study, contrary to most of the China strains, the 3-nt (TAA) deletion at position 19,471~19,473 of the S gene was not present in the CHN/GX/1468B/2017 strain as well as in the strains from the USA, Japan, and South Korea. This deletion was located in the S1 subunit which is responsible for the binding of viral receptors (15). In addition, studies have shown that the presence of a 6-nt (at position 1,733~1,738) and 9-nt deletions (at position 2,804~2,812) in the ORF1a gene is general among all the Southeast Asia strains (25). In our study, the CHN/GX/1468B/2017 strain was shown to have both deletions in the ORF1a gene, which is different to the strains from USA, Japan, Korea, and most China strains. These two deletions of ORF1a encoded Nsp2 and Nsp3 proteins, respectively (35, 36). As opposed to most other Chinese strains, the CHN/GX/1468B/2017 strain has an 8-nt deletion in the 3′UTR region at position 25,040~25,047. However, whether these particular deletions contribute to the efficiency of viral replication and virulence is subject to further study.

Compared to the Southeast Asia strain, the CHN/GX1468B/2017 strain shares the same deletions in the ORF1a gene (28), and it shows the highest homology and the closest genetic relationship to that virus. This indicates that the CHN/GX1468B/2017 strain from Guangxi, China, and the PDCoV strains from Southeast Asia might be derived from common evolutionary ancestor. Recent studies have indicated that there may been some recombination events between the PDCoV strains from China and Southeast Asia (25, 29). South-east Asia has been one of China's important trading partner and the swine production in that region is growing rapidly. The pig populations have at least doubled since 1990 in many South-East Asian countries, including Vietnam (37). In this study, no recombination event was seen in the PDCoV CHN/GX/1468B/2017 strain, but this has to be monitored carefully in future.

To evaluate the pathogenicity, 7-day-old piglets were orally inoculated with the CHN/GX/1468B/2017 strain. Different infectious doses of PDCoV can cause diarrhea in piglets, but the severity and duration of symptoms will be affected directly by the dose used (31, 32). The maximum experimental dose of infection used in these studies were limited to seven DPI in an effort to adequately capture gross and microscopic lesions caused by PDCoV infections (7, 8). It would be interesting to investigate the persistence of the virus by extending the duration of the study.

Consistent with previous reports (38, 39), severe diarrhea was observed at 36~144 HPI in the inoculated piglets and all PDCoV inoculated animals exhibited high viral shedding in their feces at 2~6 DPI. Combined with the results of PDCoV RNA detection in the oral and nasal swabs from inoculated piglets, increasing the number of experimental groups, including different routes of administration as well as an indirect contact group, would help us to confirm that the virus could be transmitted through the air (40). These possibilities will be explored in future studies.

Previous studies showed that transient viremia can occur in the early and acute stages of PDCoV infection (41). In this study, viral RNA was detected in serum from all four infected piglets at seven DPI, but it remains unclear whether the infectious virus was still present. This needs to be confirmed by identification of the virus in peripheral blood mononuclear cells which will also be explored in future studies.

The gross pathology of the small intestines from infected piglets were thin, transparent and filled with yellow watery fluid. Pathological sections showed that the small intestinal villi were severely atrophied, blunted, and the intestinal epithelial cells were swollen. In addition, the VH:CD ratios of the jejunum and ileum in piglets inoculated with the CHN/GX/1468B/2017 strain were significantly lower than the negative control group. Different levels of PDCoV genome copies were detected in other tissues, including the hearts, livers, spleens, lungs, kidneys, mesenteric lymph nodes, tonsils, and blood from infected piglets at seven DPI. Among them, the highest levels of viral RNA copies were found in the large intestines, followed by small intestines and stomachs. These results indicated that the CHN/GX/1468B/2017 strain was pathogenic in 7-day-old piglets with a wide range of tissue tropism, and the intestinal tract was the main target of PDCoV.

Most types of PDCoV cause severe intestinal disease in pigs of different ages, and the severity of the disease in pigs is age-dependent (29, 31, 39), and virus replication occurred mainly in the intestines of infected piglets where the number of viral RNA copies was the highest. It should be noted that PDCoV replicated mainly in the jejunum and ileum until 4 DPI, from where it was transmitted to the large intestine and multiplied in the cecum and colon (38). This may explain why the large intestine had the highest viral RNA load at 7 DPI in present study. Recently, a chimeric PDCoV strain which is similar to strains from Southeast Asia and the USA was identified in Vietnam, and it was able to induce villous atrophy and small intestinal enteritis in 3-day-old pigs (28). One PDCoV strain from South Korea induced acute enteritis and had the ability to induce an effective immune response in newborn piglets (42). Here, we report the full-length genome characterization and pathogenicity of a Southeast Asia-like PDCoV strain isolated from China.

To date, all the members of the *Deltacoronavirus* genus, except PDCoV, have been detected in birds (43), and PDCoV may cross the species barrier from avian/birds to some mammals (15). It was demonstrated that PDCoV has the multi-host potential to infect birds (44), chicken and calves *in vivo* (45), and to infect human cells *in vitro*. Pigs can act as intermediate hosts for multiple zoonotic viruses, and therefore, the cross-species transmission of PDCoV should be further evaluated (46).

In summary, a Southeast Asia-like PDCoV strain, CHN/GX/1468B/2017, was successfully isolated and the entire genome sequence was obtained. Analysis of the sequence alignment and genetic evolution showed that the CHN/GX/1468B/2017 strain was closely related to Southeast Asia strains. Infection of 7-day-old piglets demonstrated that the CHN/GX/1468B/2017 strain is enteropathogenic and mainly infects the gastrointestinal tracts. This study enriches the information on the genetic characteristics of PDCoV in different countries, as well as its pathogenicity in piglets.

## Data Availability Statement

The datasets presented in this study can be found in online repositories. The names of the repository/repositories and accession number(s) can be found below: https://www.ncbi.nlm.nih.gov/genbank/ (MN025260.1).

## Ethics Statement

The animal study was reviewed and approved by Animal Care and Welfare Committee of Guangxi University.

## Author Contributions

HW, YQ, WZ, and KO: conceptualization. HW, YQ, TY, ChY, XM, PZ, YL, BL, ZC, YH, CuY, XY, ZhW, YC, ZuW, and WH: data curation. HW, YQ, WZ, and KO: formal analysis. WZ and KO: funding acquisition, project administration, and supervision. YQ, WZ, and KO: resources. HW, YQ, and WZ: writing—original draft. KO: writing—review and editing. All authors contributed to the article and approved the submitted version.

## Conflict of Interest

The authors declare that the research was conducted in the absence of any commercial or financial relationships that could be construed as a potential conflict of interest.
