# Supplementary figures and images for: Genetic Characteristics and Pathogenicity of a Novel Porcine Deltacoronavirus Southeast Asia-Like Strain Found in China
Source: Front Vet Sci. 2021 Jul 16;8:701612. doi: 10.3389/fvets.2021.701612 (PMC8322666; doi:10.3389/fvets.2021.701612)

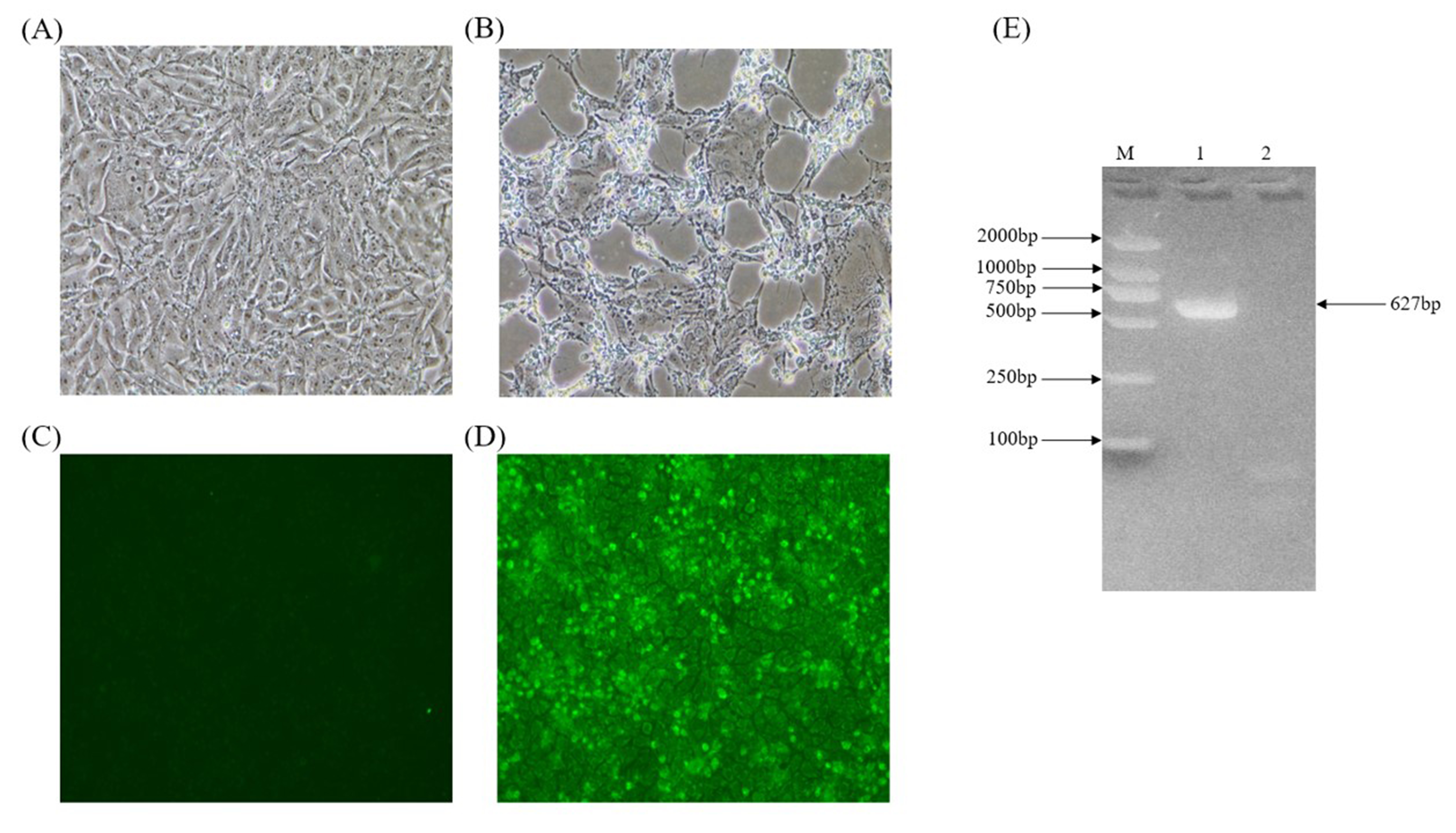

Supplement: Supplementary file 1 [file Image_1.JPEG]
