# Supplementary material for: Genetic Characteristics and Pathogenicity of a Novel Porcine Deltacoronavirus Southeast Asia-Like Strain Found in China
Source: Front Vet Sci. 2021 Jul 16;8:701612. doi: 10.3389/fvets.2021.701612 (PMC8322666; doi:10.3389/fvets.2021.701612)
Supplement: Supplementary file 3 [file Table_1.DOCX]

**Supplementary Table 1.** The sequences of the primers used in this study.

| **Primer** | **Nucleotide sequence (5′→3′)** | **Tm (℃)** | **Length (bp)** |
| --- | --- | --- | --- |
| PDCoV-F | CCAGCAACCACTCGTGTTA | 56 | 627 |
| PDCoV-R | GTCCTTAGTTGGTTTRGTRGGT |  |  |
| PEDV-F | GAAATAACCAGGGTCGTGGA | 51 | 492 |
| PEDV-R | CTCACGAACAGCCACATTA |  |  |
| TGEV-F | GATGGCGACCAGATAGAAGT | 51 | 612 |
| TGEV-R | GCAATAGGGTTGCTTGTACC |  |  |
| PoRV-F | AAAGATGCTAGGGACAAAATTG | 51 | 309 |
| PoRV-R | TTCAGATTGTGGAGCTATTCCA |  |  |
| PDCoV-QF | CCAGCAACCACTCGTGTTA | 60 | 146 |
| PDCoV-QR | GTGATTGAGTARGAGAAGGTAAGG |  |  |
| PDCoV-Probe | FAM-ATGTGCCTGGTGTTCAGGAAATGC-BHQ1 |  |  |

**Supplementary Table 2. Information regarding the reference strains of PDCoV.**

| Strain name | GenBank accession NO. | Collected date | Collected Country | Genomic length (nt) |
| --- | --- | --- | --- | --- |
| CHN/HNZK-02/2018 | MH708123.1 | 2018 | China | 25453 |
| CHN/GX/1468B/2017 | MN025260.1 | 2017 | China | 25399 |
| CHN/GS/1/2017 | MF642324.1 | 2017 | China | 25420 |
| CHN/HeB1/2017 | MG242062.1 | 2017 | China | 25414 |
| CHN/QH/1/2017 | MF642325.1 | 2017 | China | 25420 |
| CHN/GD/2017 | MF431742.1 | 2017 | China | 25420 |
| CHN/HB-BD/2017 | MF948005.1 | 2017 | China | 25423 |
| CHN/HG/2017 | MF095123.1 | 2017 | China | 25399 |
| CHN/GD/2016 | MF280390.1 | 2016 | China | 25402 |
| CHN/GS/1/2016 | MF642322.1 | 2016 | China | 25420 |
| CHN/HN/1601/2016 | MG832584.1 | 2016 | China | 25419 |
| CHN/JXJGS01/2016 | KY293677.1 | 2016 | China | 25438 |
| CHN/SHJS/SL/2016 | MF041982.1 | 2016 | China | 25414 |
| CHN/CH-01/2016 | KX443143.2 | 2016 | China | 25404 |
| CHN/NH/2016 | KU981059.1 | 2016 | China | 25420 |
| CHN/Tianjin/2016 | KY065120.1 | 2016 | China | 25413 |
| CHN/SC/2015 | MK355396.1 | 2015 | China | 25403 |
| CHN/CHJXNI2/2015 | KR131621.1 | 2015 | China | 25438 |
| CHN/SXD1/2015 | KT021234.1 | 2015 | China | 25419 |
| CHN/HKU15-S579N/2014 | LC216914.1 | 2014 | China | 25422 |
| CHN/HKU15-S582N/2014 | LC216915.1 | 2014 | China | 25406 |
| CHN/Hunan/2014 | KY513724.1 | 2014 | China | 25413 |
| CHN/Jiangsu/2014 | KY513725.1 | 2014 | China | 25422 |
| CHN/LYG/2014 | KU665558.1 | 2014 | China | 25370 |
| CHN/SD/2014 | MF431743.1 | 2014 | China | 25414 |
| CHN/HB/2014 | KP757891.1 | 2014 | China | 25420 |
| CHN/HN/2014 | KT336560.1 | 2014 | China | 25420 |
| CHN/JS/2014 | KP757892.1 | 2014 | China | 25420 |
| CHN/Sichuan/S27/2012 | KT266822.1 | 2012 | China | 25404 |
| CHN/HKU15-155/2010 | JQ065043.2 | 2010 | China | 25425 |
| CHN/HKU15-44/2009 | JQ065042.2 | 2009 | China | 25430 |
| CHN/AH/2004 | KP757890.1 | 2004 | China | 25420 |
| Lao/P1-16-BTL-0115/2016 | KX118627.1 | 2016 | Southeast | 25405 |
| Thailand/S5011/2016 | KU051641.1 | 2016 | Southeast | 25405 |
| Vietnam/Binh21/2015 | KX834352.1 | 2015 | Southeast | 25406 |
| Vietnam/HaNoi6/2015 | KX834351.1 | 2015 | Southeast | 25406 |
| Thailand/S5015L/2015 | KU051649.1 | 2015 | Southeast | 25405 |
| Thailand/TT_1115/2015 | KU984334.1 | 2015 | Southeast | 25403 |
| JPN/HKD/2016 | LC260045.1 | 2016 | Japan | 25359 |
| JPN/AKT/2014 | LC260038.1 | 2014 | Japan | 25362 |
| JPN/GNM-1/2014 | LC260039.1 | 2014 | Japan | 25362 |
| JPN/GNM-2/2014 | LC260040.1 | 2014 | Japan | 25362 |
| JPN/IWT/2014 | LC260041.1 | 2014 | Japan | 25362 |
| JPN/MYZ/2014 | LC260042.1 | 2014 | Japan | 25362 |
| JPN/OKN/2014 | LC260043.1 | 2014 | Japan | 25362 |
| JPN/YMG/2014 | LC260044.1 | 2014 | Japan | 25362 |
| KOR/DH2/2017 | KY354364.1 | 2017 | South Korea | 25422 |
| KOR/DH1/2016 | KY354363.1 | 2016 | South Korea | 25422 |
| KOR/KNU16-11/2016 | KY926512.1 | 2016 | South Korea | 25419 |
| KOR/KNU16-07/2014 | KY364365.1 | 2014 | South Korea | 25422 |
| KOR/KNU14-04/2014 | KM820765.1 | 2014 | South Korea | 25422 |
| USA/Arkansas61/2015 | KR150443.1 | 2015 | USA | 25398 |
| USA/Iowa136/2015 | KX022602.1 | 2015 | USA | 25382 |
| USA/MN140/2015 | KX022603.1 | 2015 | USA | 25394 |
| USA/Nebraska137/2015 | KX022604.1 | 2015 | USA | 25382 |
| USA/IL272/2014 | KR265856.1 | 2014 | USA | 25399 |
| USA/IL273/2014 | KR265857.1 | 2014 | USA | 25394 |
| USA/Indiana453/2014 | KR265851.1 | 2014 | USA | 25394 |
| USA/Iowa459/2014 | KR265865.1 | 2014 | USA | 25394 |
| USA/MI447/2014 | KR265849.1 | 2014 | USA | 25393 |
| USA/MI448/2014 | KR265850.1 | 2014 | USA | 25394 |
| USA/MN159/2014 | KR265859.1 | 2014 | USA | 25401 |
| USA/MN292/2014 | KR265864.1 | 2014 | USA | 25395 |
| USA/MN454/2014 | KR265854.1 | 2014 | USA | 25394 |
| USA/MN455/2014 | KR265855.1 | 2014 | USA | 25394 |
| USA/Nebraska209/2014 | KR265860.1 | 2014 | USA | 25396 |
| USA/Nebraska210/2014 | KR265861.1 | 2014 | USA | 25404 |
| USA/NorthCarolina452/2014 | KR265858.1 | 2014 | USA | 25394 |
| USA/OH444/2014 | KR265862.1 | 2014 | USA | 25394 |
| USA/Ohio445/2014 | KR265863.1 | 2014 | USA | 25394 |
| USA/HKU-OH11846/2014 | KT381613.1 | 2014 | USA | 25422 |
| USA/IL/026PDV_P11/2014 | KP981395.1 | 2014 | USA | 25422 |
| USA/OhioCVM1/2014 | KJ769231.1 | 2014 | USA | 25433 |
| USA/HKU15-IL2768/2014 | KJ584355.1 | 2014 | USA | 25422 |
| USA/HKU15-IN2847/2014 | KJ569769.1 | 2014 | USA | 25422 |
| USA/HKU15-KY4813/2014 | KJ584357.1 | 2014 | USA | 25422 |
| USA/HKU15-MI6148/2014 | KJ620016.1 | 2014 | USA | 25422 |
| USA/HKU15-MI8977/2014 | KM012168.1 | 2014 | USA | 25411 |
| USA/HKU15-NE3579/2014 | KJ584359.1 | 2014 | USA | 25422 |
| USA/HKU15-OH1987/2014 | KJ462462.1 | 2014 | USA | 25422 |
| USA/HKU15-PA3148/2014 | KJ584358.1 | 2014 | USA | 25422 |
| USA/HKU15-SD3424/2014 | KJ584356.1 | 2014 | USA | 25422 |
| USA/IA8734/2014 | KJ567050.1 | 2014 | USA | 25422 |
| USA/IL121/2014 | KJ481931.1 | 2014 | USA | 25406 |
| USA/Illinois133/2014 | KJ601777.1 | 2014 | USA | 25408 |
| USA/Illinois134/2014 | KJ601778.1 | 2014 | USA | 25404 |
| USA/Ohio137/2014 | KJ601780.1 | 2014 | USA | 25404 |
| USA/MN/2013 | KR265853.1 | 2013 | USA | 25394 |

**Supplementary Table 3.** Nucleotide and amino acid identities of the CHN/GX/1468B/2017 strain compared with 87 other reference PDCoV strains from different parts of the GenBank.

| **Area** | **Nucleotide and amino acid identity (%)** | | | | | | | | | |
| --- | --- | --- | --- | --- | --- | --- | --- | --- | --- | --- |
|  |  | **Complete genome** | **ORF1a** | **ORF1a/1b** | **S** | **E** | **M** | **NS6** | **N** | **NS7** |
| China | Nucleotide | 97.0-97.9 | 96.8-98.0 | 97.2-98.1 | 95.7-96.9 | 96.0-99.6 | 97.9-99.4 | 95.4-98.2 | 96.5-98.9 | 96.3-98.8 |
|  | Amino Acid | / | 97.7-98.8 | 97.8-99.1 | 96.0-98.1 | 94.0-100 | 97.2-99.5 | 90.4-96.8 | 95.0-99.7 | 91.0-97.0 |
| Japan | Nucleotide | 96.9-97.4 | 96.7-97.2 | 97.3-97.7 | 96.0-96.7 | 98.8-99.2 | 98.6-98.9 | 97.2-97.9 | 98.2-98.6 | 98.0-98.3 |
|  | Amino Acid | / | 97.7-98.2 | 98.4-98.8 | 97.4-98.2 | 100 | 99.5 | 94.7-95.7 | 99.4-99.7 | 94.5-95.5 |
| Korea | Nucleotide | 97.1-97.5 | 96.9-97.2 | 97.4-97.6 | 95.7-96.6 | 98.8-99.2 | 98.8-98.9 | 97.5-97.9 | 97.9-98.6 | 97.5-98.5 |
|  | Amino Acid | / | 97.9-98.2 | 98.5-98.8 | 96.9-98.2 | 100 | 99.5 | 95.7-96.8 | 99.4-99.7 | 93.5-96.0 |
| Southeast Asia | Nucleotide | 98.3-99.4 | 98.8-99.4 | 98.7-99.5 | 96.7-98.9 | 99.2-99.6 | 98.6-99.8 | 97.9-98.9 | 96.8-99.5 | 96.7-99.3 |
|  | Amino Acid | / | 99.1-99.4 | 99.3-99.5 | 97.7-99.1 | 100 | 99.1-99.5 | 96.8-97.9 | 98.5-100 | 91.5-98.5 |
| USA | Nucleotide | 97.3-97.6 | 97.1-98.8 | 97.3-97.7 | 96.4-96.7 | 98.8-99.6 | 98.5-99.1 | 97.5-98.2 | 98.3-98.7 | 98.0-98.7 |
|  | Amino Acid | / | 98.0-98.2 | 98.7-98.8 | 97.7-98.3 | 100 | 99.1-99.5 | 94.7-96.8 | 99.1-99.7 | 94.5-97.0 |

**Supplementary Figure Legends**

**Supplementary Figure. 1. Isolation and identification of the PDCoV CHN/GX/1468B/2017 strain.** (A) LLC-PK control cells. (B) Cytopathic effects of PDCoV-infected LLC-PK cells at 24 HPI. The cells were enlarged, rounded and aggregated and eventually died. (C) Negative control for IFA. (D) IFA staining in LLC-PK cells infected with the PDCoV CHN/GX/1468B/2017 strain at 24 h with mouse-derived PDCoV-N protein-specific polyclonal antibody and FITC-labeled goat anti-mouse IgG, respectively. This photomicrograph shows that the specific green fluorescence was detected in the PDCoV infected cells. Magnification = 200×. (E) RT-PCR amplification of the N gene fragment of PDCoV isolated from the small intestinal content of an animal from a swine farm in Guangxi. M: DL 2000 marker; Lane 1: predicted product (627bp); Lane 2: negative control.

**Supplementary Figure. 2. Body weight changes from piglets challenged with the PDCoV CHN/GX/1468B/2017 strain.** The weight gain trend of piglets after being challenged with the PDCoV CHN/GX/1468B/2017 strain. Body weight was measured daily. The weight gain rate of piglets in the challenge group was always lower than that of the control group, but this was not significant. Each point on the curve represents the average gain rate from four piglets.
